# *OCA2* splice site variant in German Spitz dogs with oculocutaneous albinism

**DOI:** 10.1371/journal.pone.0185944

**Published:** 2017-10-03

**Authors:** Madleina Caduff, Anina Bauer, Vidhya Jagannathan, Tosso Leeb

**Affiliations:** 1 Institute of Genetics, Vetsuisse Faculty, University of Bern, Bern, Switzerland; 2 DermFocus, University of Bern, Bern, Switzerland; University of Sydney Faculty of Veterinary Science, AUSTRALIA

## Abstract

We investigated a German Spitz family where the mating of a black male to a white female had yielded three puppies with an unexpected light brown coat color, lightly pigmented lips and noses, and blue eyes. Combined linkage and homozygosity analysis based on a fully penetrant monogenic autosomal recessive mode of inheritance identified a critical interval of 15 Mb on chromosome 3. We obtained whole genome sequence data from one affected dog, three wolves, and 188 control dogs. Filtering for private variants revealed a single variant with predicted high impact in the critical interval in *LOC100855460* (XM_005618224.1:c.377+2T>G LT844587.1:c.-45+2T>G). The variant perfectly co-segregated with the phenotype in the family. We genotyped 181 control dogs with normal pigmentation from diverse breeds including 22 unrelated German Spitz dogs, which were all homozygous wildtype. Comparative sequence analyses revealed that *LOC100855460* actually represents the 5’-end of the canine *OCA2* gene. The CanFam 3.1 reference genome assembly is incorrect and separates the first two exons from the remaining exons of the *OCA2* gene. We amplified a canine *OCA2* cDNA fragment by RT-PCR and determined the correct full-length mRNA sequence (LT844587.1). Variants in the *OCA2* gene cause oculocutaneous albinism type 2 (OCA2) in humans, pink-eyed dilution in mice, and similar phenotypes in corn snakes, medaka and Mexican cave tetra fish. We therefore conclude that the observed oculocutaneous albinism in German Spitz is most likely caused by the identified variant in the 5’-splice site of the first intron of the canine *OCA2* gene.

## Introduction

Oculocutaneous albinism (OCA) summarizes a group of inherited disorders of melanin synthesis, characterized by hypopigmentation in skin, hair and eyes [1]. Six of the seven types of human OCA are assigned to variants in a specific gene: OCA1 (*TYR*), OCA2 (*OCA2*), OCA3 (*TYRP1*), OCA4 (*SLC45A2*), OCA6 (*SLC24A5)* and OCA7 (*LRMDA*) [1,2]. Variants in *OCA2* cause OCA2 in humans (OMIM #203200) and pink-eyed dilution in the mouse [3]. Up to 150 human and 100 murine *OCA2* variants have been reported [4,5]. Additionally, amelanism in corn snakes and albinism in medaka and Mexican cave tetra fish are also caused by variants in *OCA2* [6–8].

The phenotype in human OCA2 patients is variable. The amount of residual cutaneous pigment varies and typically increases with age. Iris color is also variable. As with other types of oculocutaneous albinism, hypopigmentation of the iris leading to iris translucency, reduced pigmentation of the retinal pigment epithelium, foveal hypoplasia, reduced visual acuity, refractive errors, and sometimes a degree of color vision impairment may occur. The human OCA2 phenotype is the most common type of OCA in Africans and has also been termed brown OCA in Africans [1,2].

The human *OCA2* gene spans 380 kb on chromosome 15 and the major transcript isoform contains 24 exons [9]. The OCA2 melanosomal transmembrane protein, formerly called P-protein, is a putative anion transporter with 12 transmembrane domains. OCA2 plays a role in melanosome biogenesis, melanosomal pH regulation, and eumelanin synthesis [3,5,10,11]. It is required for the normal processing and transport of other melanosomal proteins, such as TYR and TYRP1 [1].

The aim of the current study was to unravel the molecular genetics for an oculocutaneous albinism phenotype in a family of German Spitz dogs.

## Results

### Phenotype characterization

**Fig 1:**
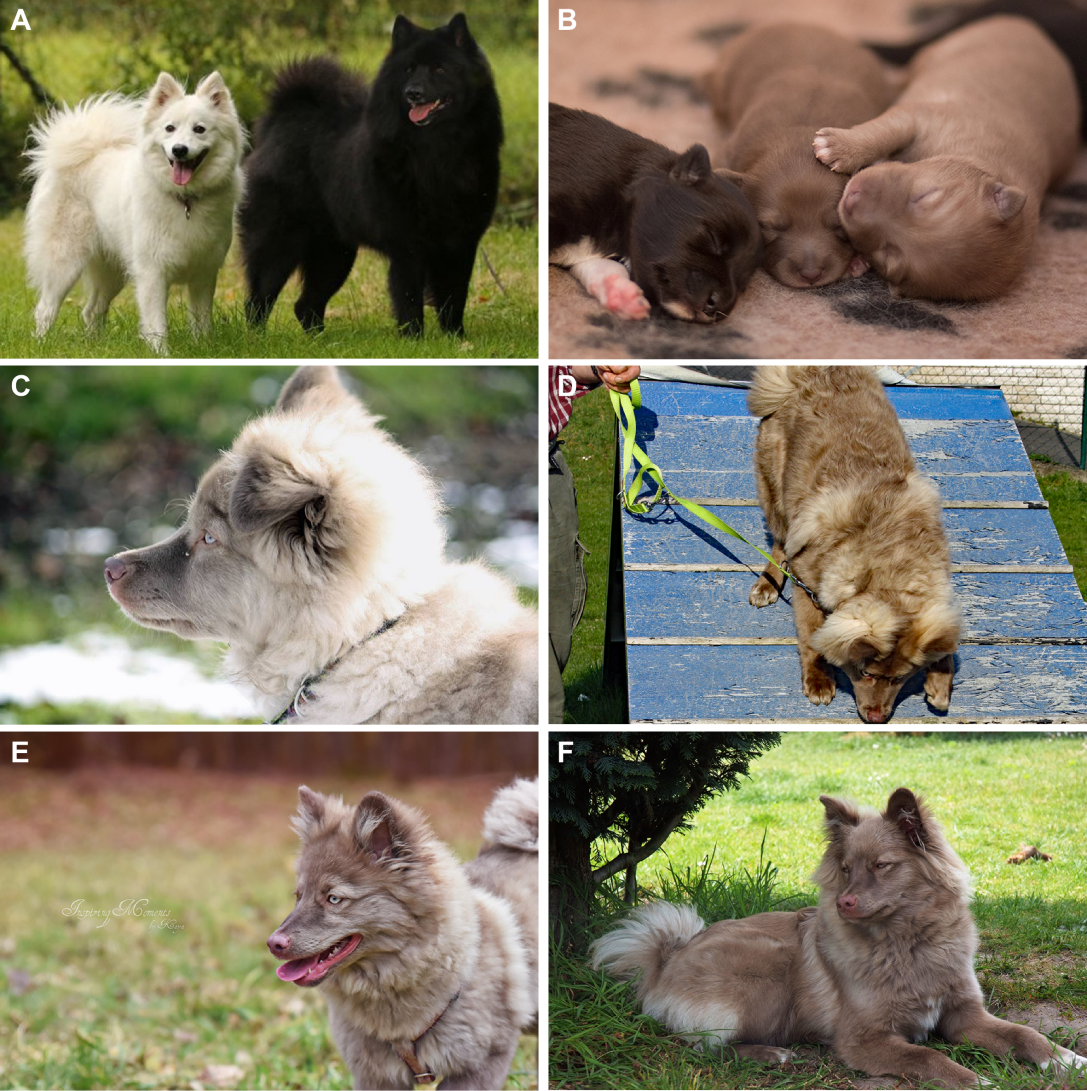
Coat color phenotypes in the investigated Giant Spitz family. **(A)** White mother and black father of the litter. (**B)** Pictures of two affected puppies and one unaffected sibling on the left at one week of age. **(C, E)** Affected dog GS103 with green eyes and a light nose at 4 and 5.5 months of age, respectively. **(D, F)** Affected dog GS104 at 4 and 7 months of age, respectively.

A German Spitz family of the Giant Spitz variety was brought to our attention where the mating of a black sire and a white bitch had produced a litter with six puppies. Three puppies had the expected black coat color, while the other three puppies, two males and one female, were of a light brown color, which could not be explained by the genotypes of the known basic coat color loci (Fig 1). Father, mother and the unaffected littermates had dark-colored noses and brown to amber eyes, whereas the three brown puppies had light lips and noses and blue eyes that turned into green with age. Dilated pupils appeared of reddish color. The coat color darkened somewhat with age. The owner reported that the affected puppies used to squint in bright sunlight (photophobia) and had difficulties to perceive hand signals in bright sunlight. Breeders’ reports indicated that dogs with similar phenotypes had previously been noticed in this line of dogs. These data suggested a monogenic autosomal recessive mode of inheritance.

### Mapping of the causative locus

**Fig 2:**
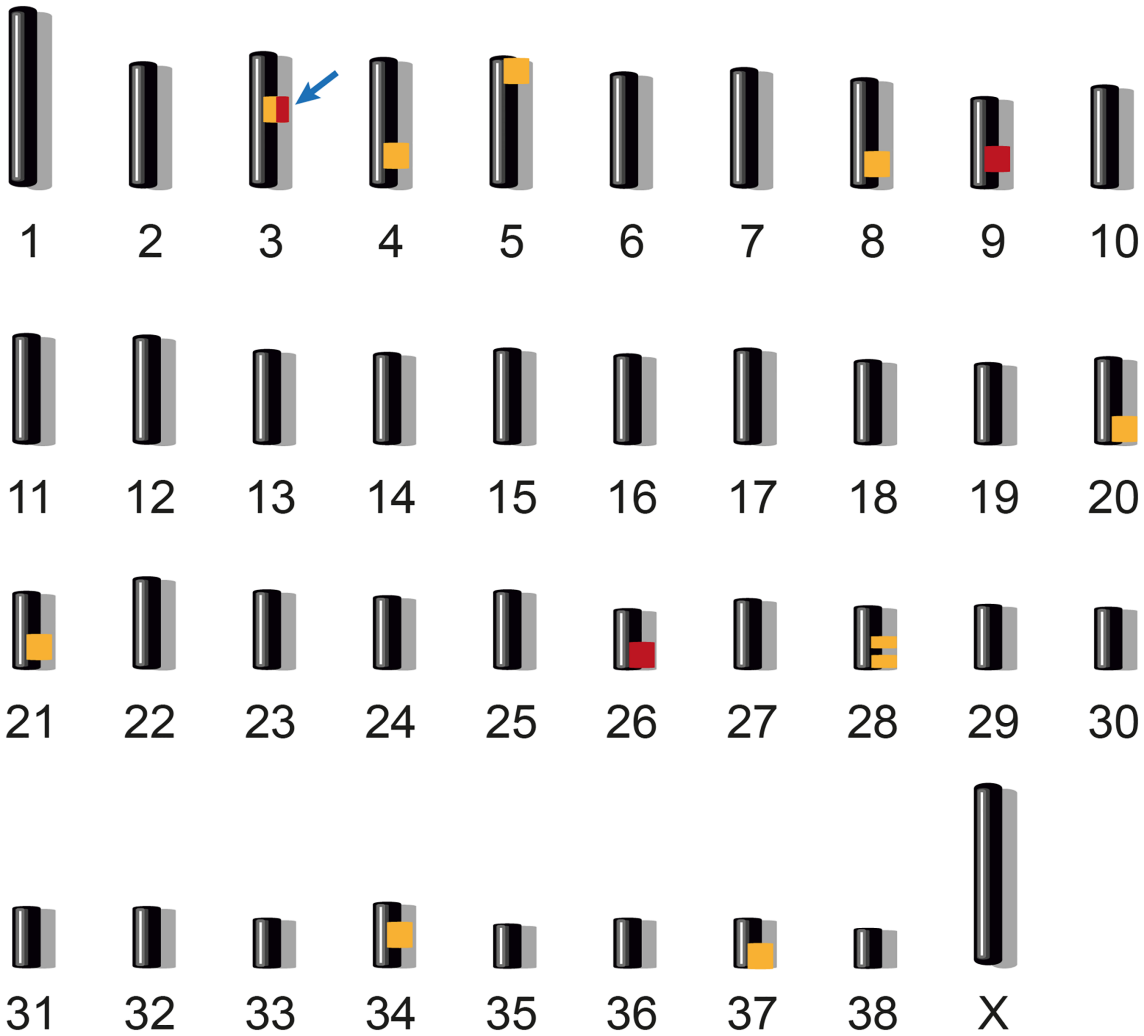
Combined linkage and homozygosity mapping. We performed parametric linkage analysis for a recessive trait in all eight family members and homozygosity mapping across the three affected German Spitz dogs. Ten linked genome segments are indicated in orange and three homozygous segments with shared alleles are indicated in red. Only one region on chromosome 3 showed both linkage and homozygosity and was considered the critical interval (arrow). Specifically, this ~15 Mb region corresponded to Chr3:28,747,944–43,731,542 (CanFam 3.1 assembly).

We genotyped all members of the family on the Illumina canineHD SNP chip and employed a combined linkage and homozygosity approach in the family with its six offspring. Parametric linkage analysis was performed assuming a fully penetrant, monogenic autosomal recessive inheritance of the trait. This analysis identified 10 segments ≥ 1 Mb that showed positive LOD scores (S1 Table). Autozygosity mapping in the three affected dogs yielded three homozygous regions with shared alleles (S2 Table). Only one genome segment, Chr3:28,747,944–43,731,542, showed both linkage and homozygosity (Fig 2).

### Identification of the causative variant

We resequenced the whole genome of one affected dog and called single nucleotide as well as indel variants with respect to the reference genome of a non-affected Boxer (CanFam 3.1). The genotypes of the affected German Spitz were further compared with 188 dog genomes from various breeds and three wolf genomes that had been sequenced in the course of other studies. We hypothesized that the causative variant should be completely absent from all other genomes except the German Spitz with oculocutaneous albinism.

Within the critical interval, this filtering process yielded only one single private variant with predicted impact on a protein-coding gene (Table 1). This variant affected the 5’-splice site of the single intron of *LOC100855460* (Chr3:31,715,704A>C; XM_005618224.1:c.377+2T>G).

**Table 1:** Variants detected by whole genome resequencing of an affected German Spitz.

Filtering step	Number of variants
Variants in the whole genome^a^	540,127
Variants in the critical 15 Mb interval on chromosome 3	5,855
Variants in the critical interval that were absent from 191 other dog genomes	199
Protein-changing variants in the whole genome^a^	2,317
Protein-changing variants in the 15 Mb critical interval on chromosome 3	15
Protein-changing variants in the critical interval, absent from 191 other dog genomes	1

^a^The sequences were compared to the reference genome (CanFam 3.1) from a Boxer. Only variants that passed GATK quality filters and were homozygous in the affected German Spitz GS104 are reported. Protein-changing variants were classified based on the NCBI annotation release 104.

**Fig 3:**
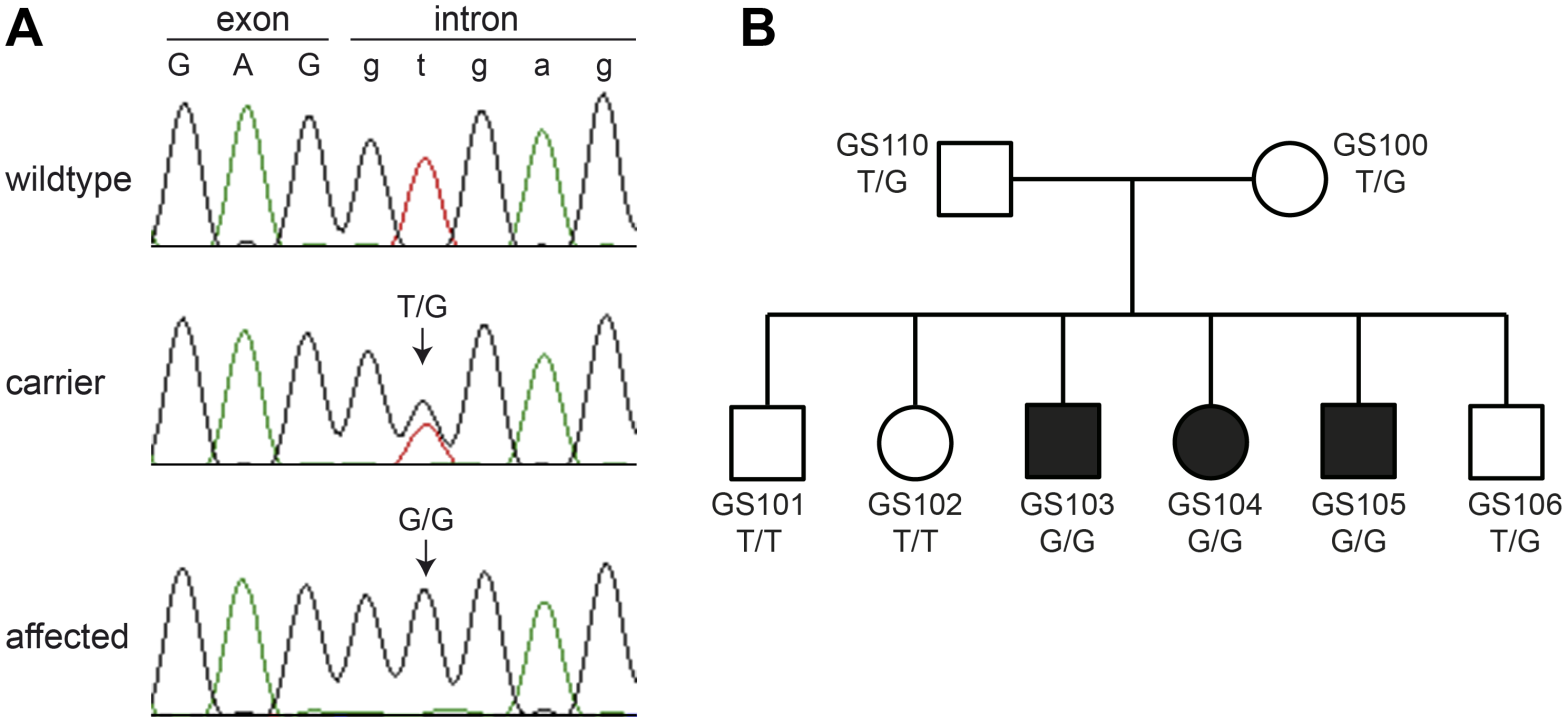
Genotypes of the German Spitz litter. **(A)** Sanger sequencing chromatograms illustrate sequences of a homozygous wildtype dog, a heterozygous carrier and a homozygous affected dog. The variant replaced the essential thymine at position +2 of the 5’-splice site with a guanine. **(B)** Pedigree of the Giant Spitz litter. Genotypes at the splice site variant show the expected co-segregation with the phenotype.

We confirmed the splice site variant by Sanger sequencing and genotyped the 8 family members, 22 unrelated German Spitz dogs (8 Giant Spitz dogs, 7 Miniature Spitz dogs and 7 Pomeranians), and 159 control dogs from various other breeds (Fig 3A). The genotypes at this variant showed perfect co-segregation with the phenotype (Fig 3B). None of the 181 normally pigmented control dogs carried the mutant allele (S3 Table).

### *LOC100855460* represents the 5’-end of the *OCA2* gene

**Fig 4:**
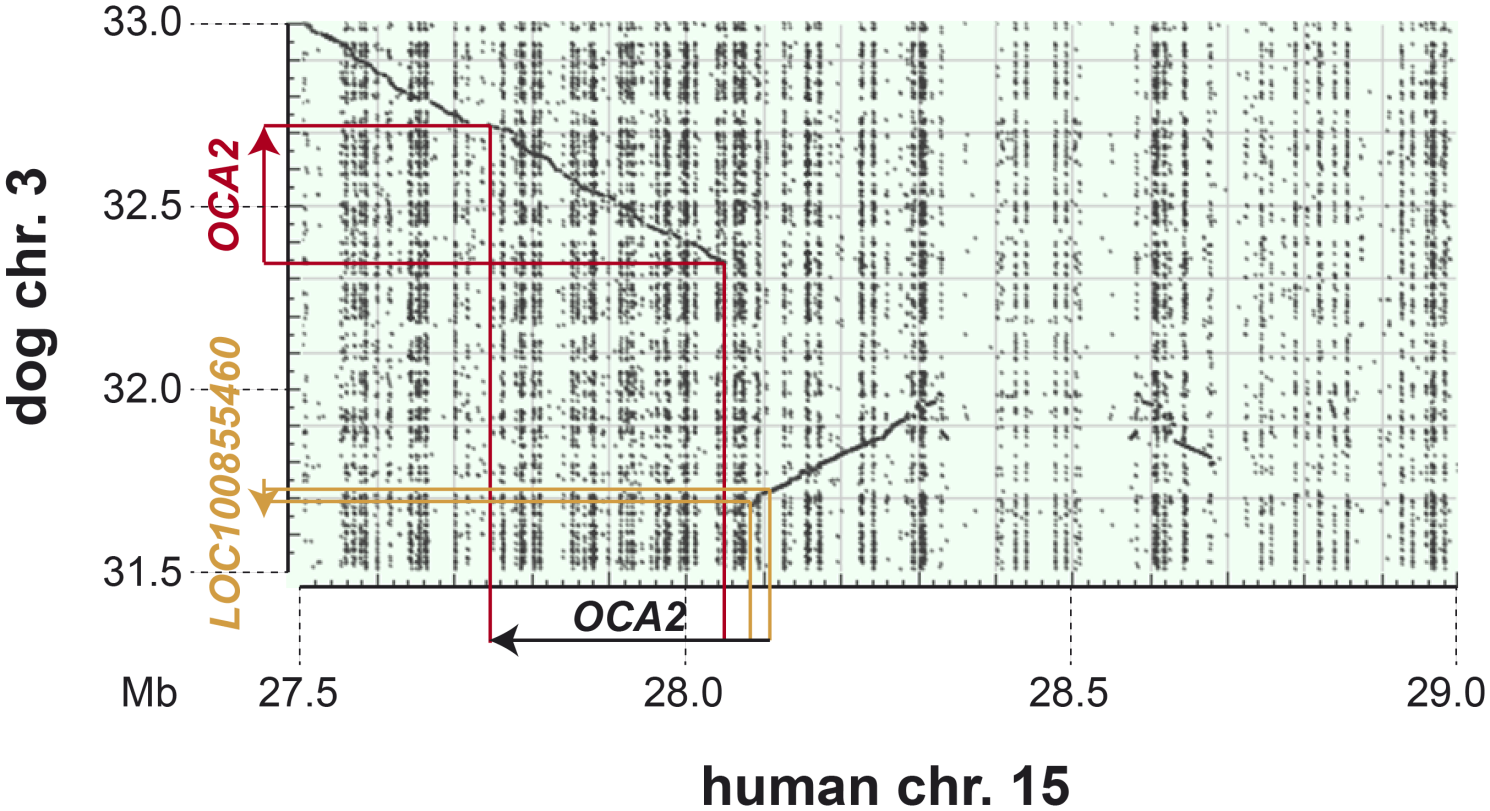
Dot plot of human Chr15:27,500,001–29,000,000 against dog Chr3:31,500,001–33,000,000. The human *OCA2* gene spans 380 kb. The dot plot illustrates that ~300 kb of dog reference sequence are inverted with respect to the human sequence due to an assembly error in the current CanFam 3.1 assembly. Due to this assembly error the first two exons of the canine *OCA2* gene are currently in the wrong orientation and annotated as *LOC100855460*.

As the *LOC100855460* splice site variant represented our best candidate causative variant for the observed oculocutaneous albinism phenotype, we performed BLAST searches with *LOC100855460* to identify putative homologs in other mammalian species. These analyses revealed that *LOC100855460* corresponds to the first two exons of the human *OCA2* gene. The currently incorrect canine gene annotation in the region is most likely due to an assembly error in the CanFam3.1 reference genome that contains a ~300 kb segment of genomic DNA in inverted orientation (Fig 4).

### Experimental confirmation of the proposed canine *OCA2* gene structure

We confirmed the suspected genome assembly error by long-range PCR on genomic DNA. We then designed an RT-PCR primer pair with a forward primer in *LOC100855460* and a reverse primer in the canine *OCA2* gene. RT-PCR and subsequent Sanger sequencing of the product demonstrated that *LOC100855460* and *OCA2* are indeed parts of the same transcript.

Based on the RT-PCR results and public RNA-seq data, we determined a full-length canine *OCA2* mRNA sequence and submitted it to the European Nucleotide Archive (accession number LT844587.1). This transcript contains an open reading frame of 2,532 nucleotides encoding a protein of 844 amino acids that shows 83% identity to the 838 amino acid human OCA2 melanosomal transmembrane protein, isoform 1 (NP_000266.2). Most of the sequence differences are located in the first 180 residues at the N-terminus.

Based on our revised canine *OCA2* full-length sequence, the candidate causative variant for oculocutaneous albinism in dogs should be designated *OCA2*:LT844587.1:c.-45+2T>G. It affects the 5’-splice site of the first intron of the canine *OCA2* gene.

## Discussion

In this study, we identified a splice site variant in the canine *OCA2* gene as most likely cause for an oculocutaneous albinism phenotype in dogs. There are two main arguments supporting the claimed causality: The variant affects the conserved GT dinucleotide at the 5’-splice site of an AG-GT intron [12]. Such a genomic alteration is not compatible with the normal splicing process and is predicted to lead to a loss of function of the mutant gene. Secondly, we provided evidence that the splice site variant affects the first intron of the canine *OCA2* gene. The functional role of this gene in pigmentation is well known and numerous genetic variants in humans, mice, and other vertebrates, lead to oculocutaneous albinism phenotypes that are very similar to the phenotypes observed in the dogs in this study [3–8]; (OMIA 000202–7994, OMIA 000202–94885). *OCA2* mutant alleles were reported to mainly block eumelanin synthesis with a less pronounced effect on pheomelanin synthesis [4]. Humans with OCA2 show a dramatic reduction of eumelanin in skin, eye and hair, resulting in cream-colored skin and yellow-straw hair, which darkens with age [13]. Melanosomes with an irregular shape were observed in p-deficient mice [14], and measurements of melanin content revealed a great decrease of eumelanin, but not pheomelanin levels [15,16]. *OCA2* expression was only found in the black dorsal, but not in the yellow ventral part of black-and-tan (*a*^*t*^*/a*^*t*^) mice, whereas it was present in the ventral black skin of non-agouti (*a/a*) mice [3]. These findings are in line with the phenotype of the *OCA2* deficient dogs in the present study. Based on their genotypes at the other coat color loci, the three affected dogs would have been expected to be black due to the presence of the dominant *K*^*B*^ allele (*E/e*; *A*^*w*^*/a*; *K*^*B*^*/k*^*y*^; *B/B*, *D/-*). It is not clear whether the residual light brown pigment in the affected dogs represents a mixture of eumelanin and pheomelanin or whether this is an abnormal melanin with a brownish color.

Accumulation of pigment with age has been described in human OCA2 patients [17] and was observed in the affected dogs as the eye color changed from blue into light green and the coat color darkened. The observed photophobia and translucent irises of the affected dogs agrees with the description of visual abnormalities in human OCA patients [1,18]. Due to the photophobia, such dogs should not be bred on purpose and carrier x carrier matings should be avoided in the future.

This study highlights the current status of genomic resources in dogs and many other domestic animal species. Although whole genome sequencing has been shown to represent a powerful technology when it comes to identifying causative variants for Mendelian traits, the bioinformatic data analysis is hindered by imperfect reference genome assemblies and gene annotations. With the current resources, a significant amount of human expert data interpretation is required. In the future, with the availability of better reference genomes, it may be expected that similar analyses will become easier and involve a greater extent of automated data analysis.

In conclusion, we identified a variant in the conserved 5’-splice site of the first intron of the canine *OCA2* gene as most likely cause for the observed oculocutaneous albinism in a Giant Spitz family. This will facilitate genetic testing to avoid the accidental breeding of further puppies with this phenotype. Our work has revealed an assembly error in the CanFam3.1 assembly and we provide a revised full-length sequence of the canine *OCA2* cDNA.

## Materials and methods

### Ethics statement

All animal experiments were performed according to the local regulations. The dogs in this study were examined with the consent of their owners. The study was approved by the “Cantonal Committee For Animal Experiments” (Canton of Bern; permits 75/16 and 38/17).

### DNA samples and genotyping

We obtained EDTA-blood samples from both parents and all 6 puppies of the German Spitz litter (Giant Spitz variety). We isolated genomic DNA with the Maxwell RSC Whole Blood DNA Kit and the Maxwell RSC Instrument (Promega). Genotyping of the two parents and six littermates was performed by GeneSeek/Neogen on the Illumina CanineHD BeadChip containing 220,853 markers. We also used 181 canine control DNA samples from the Vetsuisse Biobank that had been collected during the course of other projects.

### Linkage analysis and homozygosity mapping

The genotype data of the 8 family members was used for a parametric linkage analysis. The call rate was > 95% for all dogs. Using PLINK v 1.07 [19], markers that were non-informative, located on the sex chromosomes, or missing in any of the 8 dogs, had Mendel errors, or a minor allele frequency < 0.3125, were removed. The final pruned dataset contained 34,356 markers. An autosomal recessive inheritance model with full penetrance, a disease allele frequency of 0.5 and the Merlin software [20] were applied to test for linkage. Intervals with positive LOD scores and α = 1 were retained for further analysis.

For homozygosity mapping, the genotype data of the three affected dogs were used. Markers that were missing in one of the three cases, markers on the sex chromosomes and markers with Mendel errors in the family were excluded. Using the—homozyg and—homozyg-group options in PLINK, extended regions of homozygosity > 1 Mb were identified. The homozygous intervals in the three cases were intersected with the linked intervals to define minimal critical intervals.

### Whole-genome sequencing

An Illumina TruSeq PCR-free library with an insert size of 350 bp was prepared from one affected dog (GS104). The library was sequenced at 32x genome coverage using 2 x 150 bp reads on an Illumina HiSeq 3000 instrument. Single nucleotide and small indel variants with respect to the CanFam3.1 canine reference genome assembly were called as described [21]. Variants private to GS104 were identified by filtering variants that were contained in 3 wolf and 188 control dog genomes sequenced for previous projects (S4 Table). Private variants were prioritized according to their predicted impact using SNPeff [22] and the NCBI annotation release 104.

### Sanger sequencing

We used Sanger sequencing to confirm the Illumina sequencing results and to perform targeted genotyping for the splice site variant in *LOC100855460*. AmpliTaqGold360Mastermix (Applied Biosystems) and the primer pair GTCTGGCCTTTCCGTGAG (forward primer) and CGAAGCTTGTGCTCAATGTC (reverse primer) were used to amplify the region by PCR. PCR products were directly sequenced on an ABI 3730 capillary sequencer (Applied Biosystems) after treatment with exonuclease I and shrimp alkaline phosphatase. The reverse primer was used as sequencing primer. We analyzed the sequence data with Sequencher 5.1 (GeneCodes).

### Long-range PCR

A long-range PCR to was performed using the primer pair GGCAAACTTGGGAGTGGTAA (Chr3:31,659,781–31,659,762) and CCCCCTCAAATAAACCATGA (Chr3:32,346,375–32,346,356) and the SequalPrep^™^ Long PCR kit (Invitrogen). The PCR fragments were analyzed using FragmentAnalyzer^™^ (Advanced Analytical).

### RT-PCR

We isolated total RNA from a skin biopsy of a healthy control dog using QIAzol and RNeasy spin columns according to the manufacturer’s recommendations (Qiagen). RNA samples were treated with RNase-free DNase to remove contaminations with genomic DNA. Reverse transcription was carried out using an oligo-dT primer, and Superscript^®^ IV Reverse Transcriptase according to the manufacturer’s recommendations (Invitrogen). PCR and sequencing were performed with 2 μl of the synthesized cDNA and the primer pair TTCTTTCTGGCTGACCTCGT (forward primer, *OCA2* exon 2, Chr3:31,681,124–31,681,105) and ATGCACCATGACCCTTTCTC (reverse primer, *OCA2* exons 4 & 3, Chr3:32,366,619–32,366,608 & Chr3:32,362,337–32,362,330), using the forward primer as a sequencing primer. For determination of the 5’- and 3’-ends of the *OCA2* mRNA sequence, we utilized publicly available RNA-seq data from canine nasal skin (ENA project accession PRJEB14109, sample accession SAMEA4412813, Lab ID LA1666).

## Supporting information

S1 Table Results of linkage analysis. (XLSX)

S2 Table Results of homozygosity mapping. (XLSX)

S3 Table Genotypes of control dogs. (XLSX)

S4 Table Accession numbers of 192 dog/wolf genome sequences. (XLSX)
